# Supplementary material for: Antibacterial effects of nanopillar surfaces are mediated by cell impedance, penetration and induction of oxidative stress
Source: Nat Commun. 2020 Apr 2;11:1626. doi: 10.1038/s41467-020-15471-x (PMC7118135; doi:10.1038/s41467-020-15471-x)
Supplement: Supplementary file 1 — Supplementary Information [file 41467_2020_15471_MOESM1_ESM.pdf]

Supplementary Information

Antibacterial effects of nanopillar surfaces are mediated by cell impedance, penetration and induction of oxidative stress

Jenkins et al.

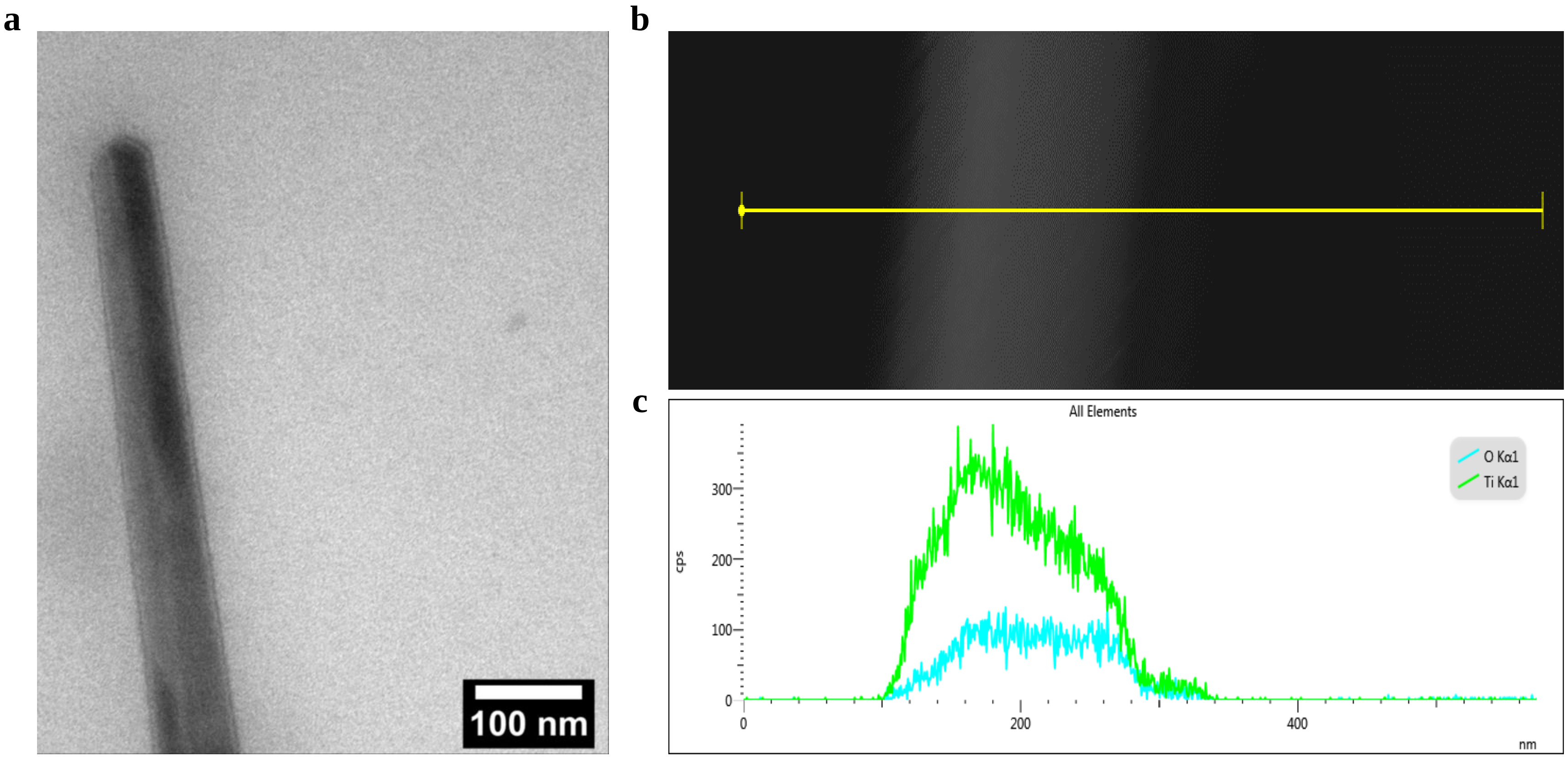

Supplementary Figure 1 | Elemental analysis of nanopillars. Single nanopillar visualised under bright-field TEM (a). EDX-line scanning across a single nanopillar in dark-field mode (b) confirmed the presence of titanium (green spectra) and oxygen (blue spectra) (c). EDX analysis was performed on five nanopillars.

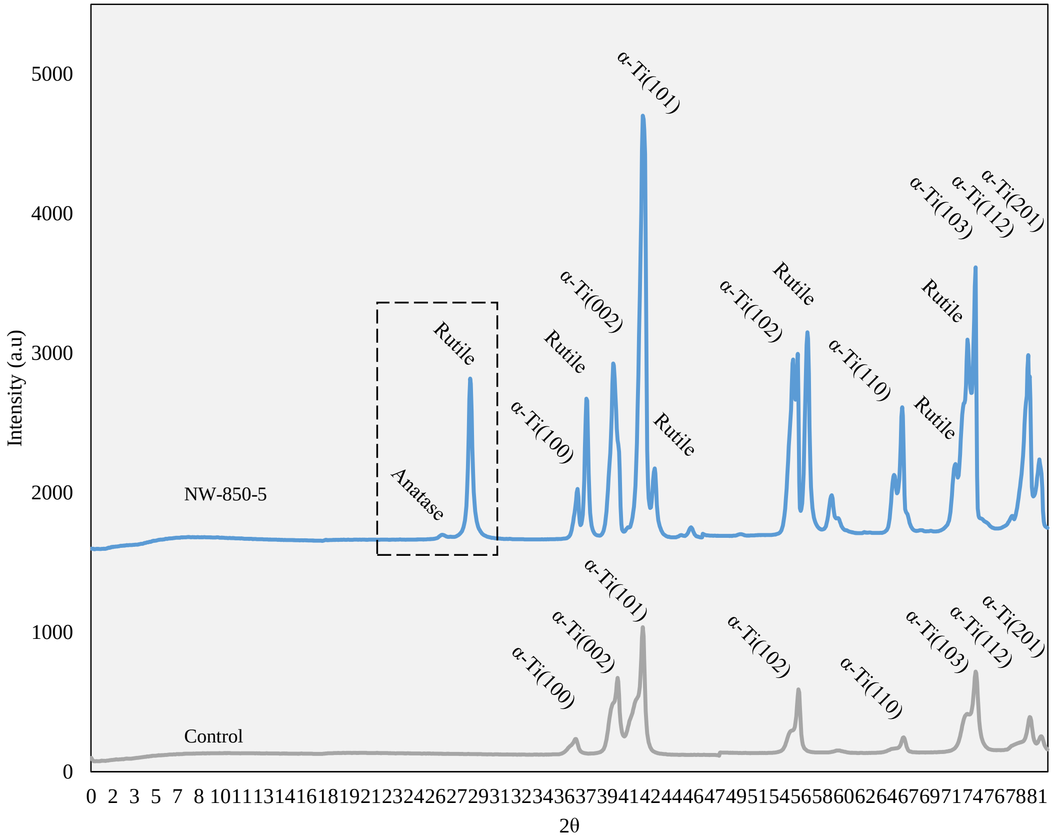

Supplementary Figure 2 │ Determination of TiO_2 nanowire crystal structure. Certain TiO_2 polymorphs (e.g. anatase) exhibit strong photocatalytic activity when exposed to ultraviolet radiation, leading to the production of free radicals that are toxic to bacterial cells. In this context, we investigated the type of TiO_2 polymorph present in nanowires to determine their photocatalytic potential. The ratio of anatase to rutile was determined using the Spurr and Myers equation[1], by calculating the area underneath characteristic anatase (2θ = 26.5) and rutile (2θ = 27.5) peaks, shown inside the dashed box. GIXD revealed that nanowires consisted mostly of rutile TiO_2 (98%) with anatase polymorphs present at much lower quantities (2%).

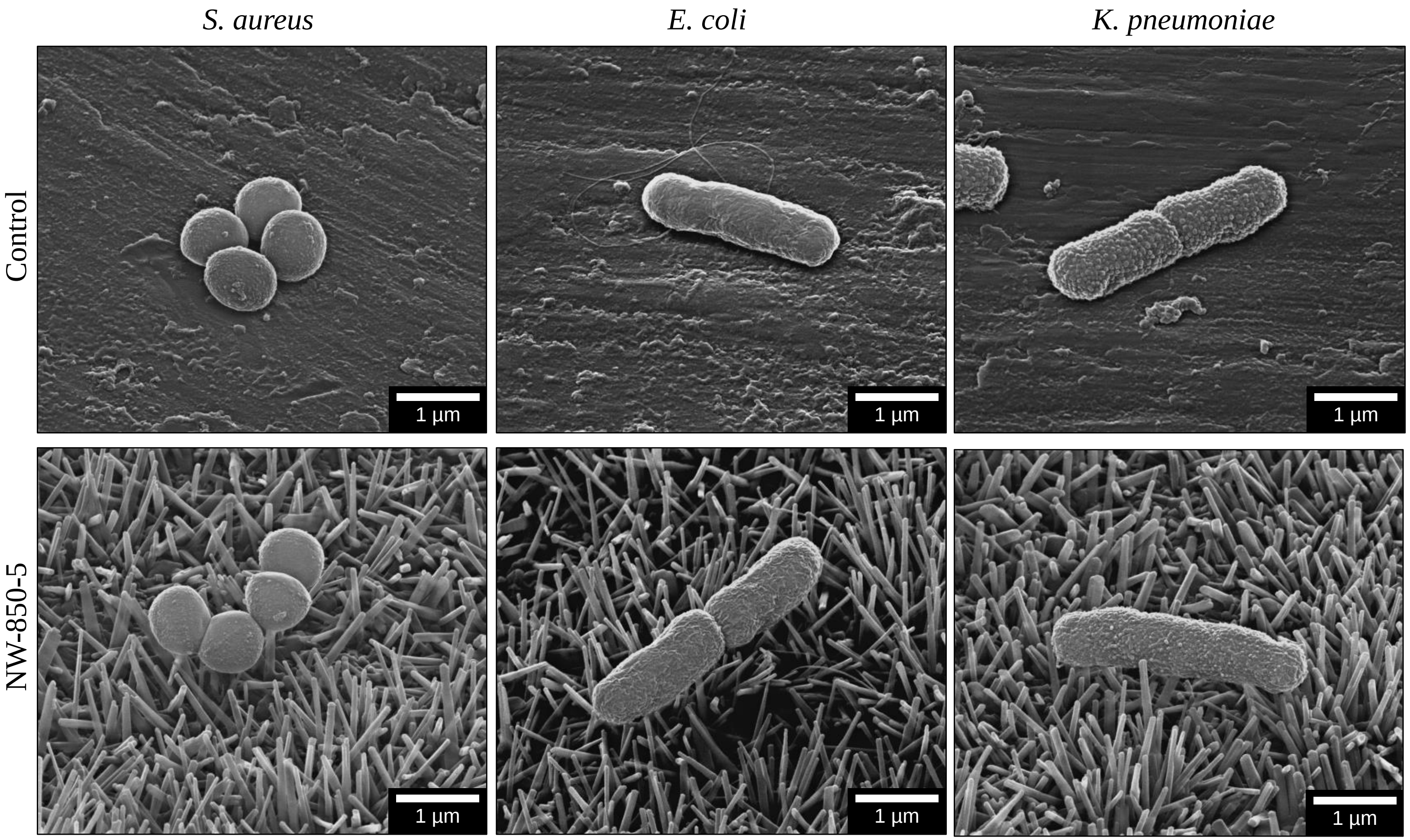

Supplementary Figure 3 | Determining bacterial morphology on nanopillar surface NW-850-5 after 0.5-hour incubation. Scanning electron micrographs of Gram-positive (*S. aureus*) or Gram-negative (*E. coli*, *K. pneumoniae*) bacteria following 0.5-hour, static incubation on flat titanium alloy (control) and TiO_2 nanopillar surface NW-850-5. Micrographs are representative of three independent surfaces (n=3).

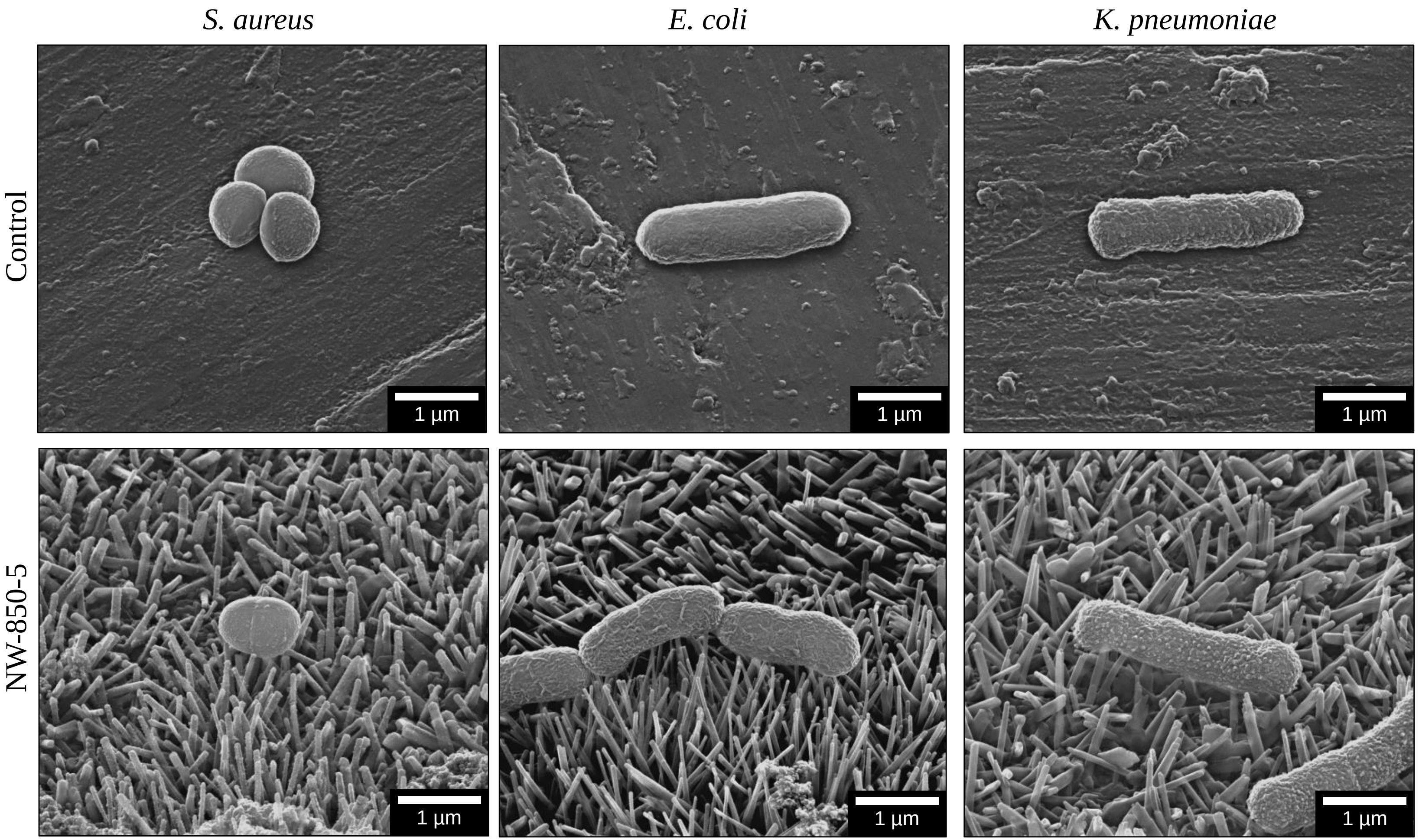

Supplementary Figure 4 | Determining bacterial morphology on nanopillar surface NW-850-5 after 1-hour incubation. Scanning electron micrographs of Gram-positive (*S. aureus*) or Gram-negative (*E. coli*, *K. pneumoniae*) bacteria following 1-hour, static incubation on flat titanium alloy (control) and TiO_2 nanopillar surface NW-850-5. Micrographs are representative of three independent surfaces (n=3).

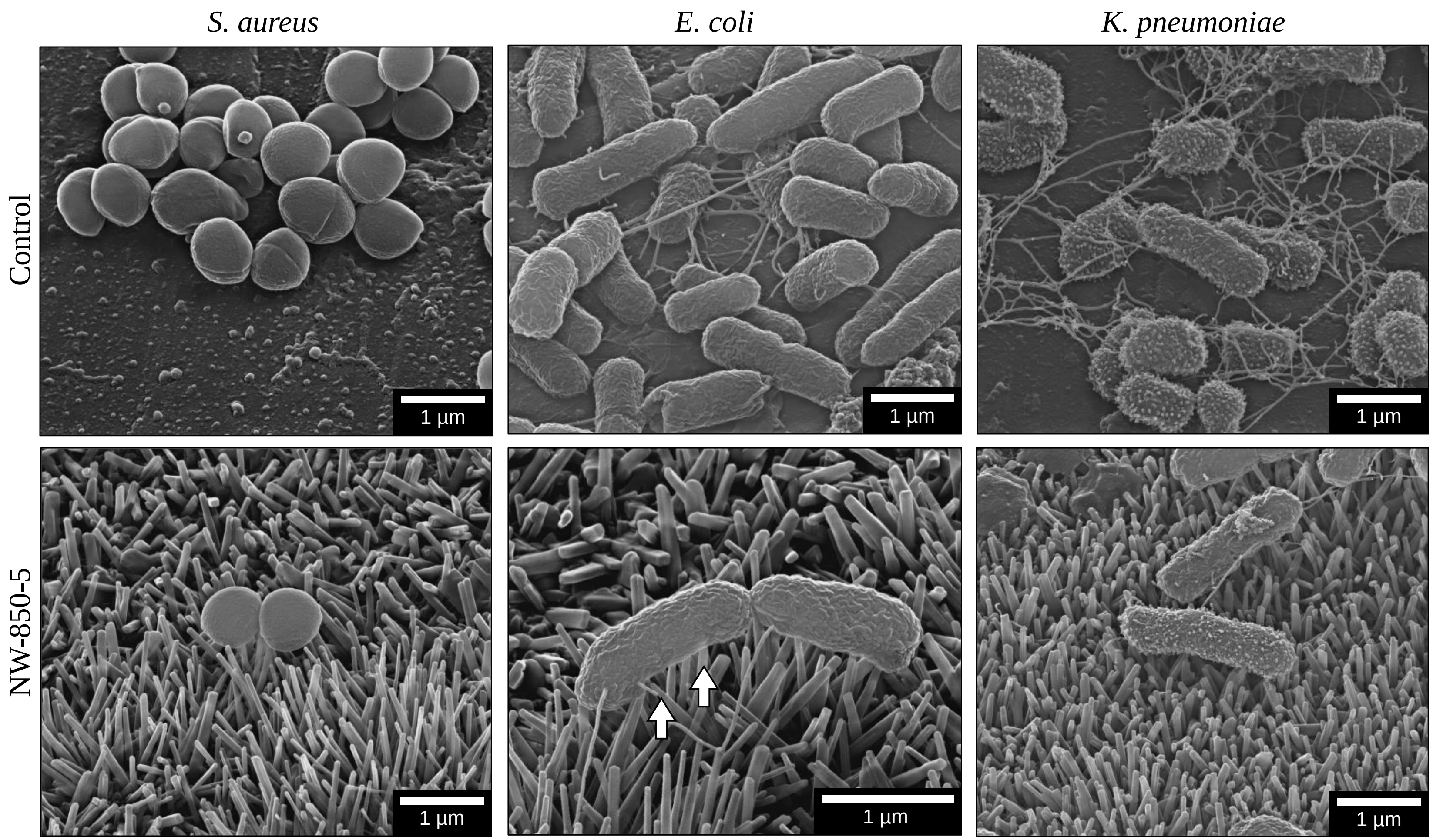

Supplementary Figure 5 | Determining bacterial morphology on nanopillar surface NW-850-5 after 10-hour incubation. Scanning electron micrographs of Gram-positive (*S. aureus*) or Gram-negative (*E. coli*, *K. pneumoniae*) bacteria following 10-hour, static incubation on flat titanium alloy (control) and TiO_2 nanopillar surface NW-850-5. Micrographs are representative of three independent surfaces (n=3).

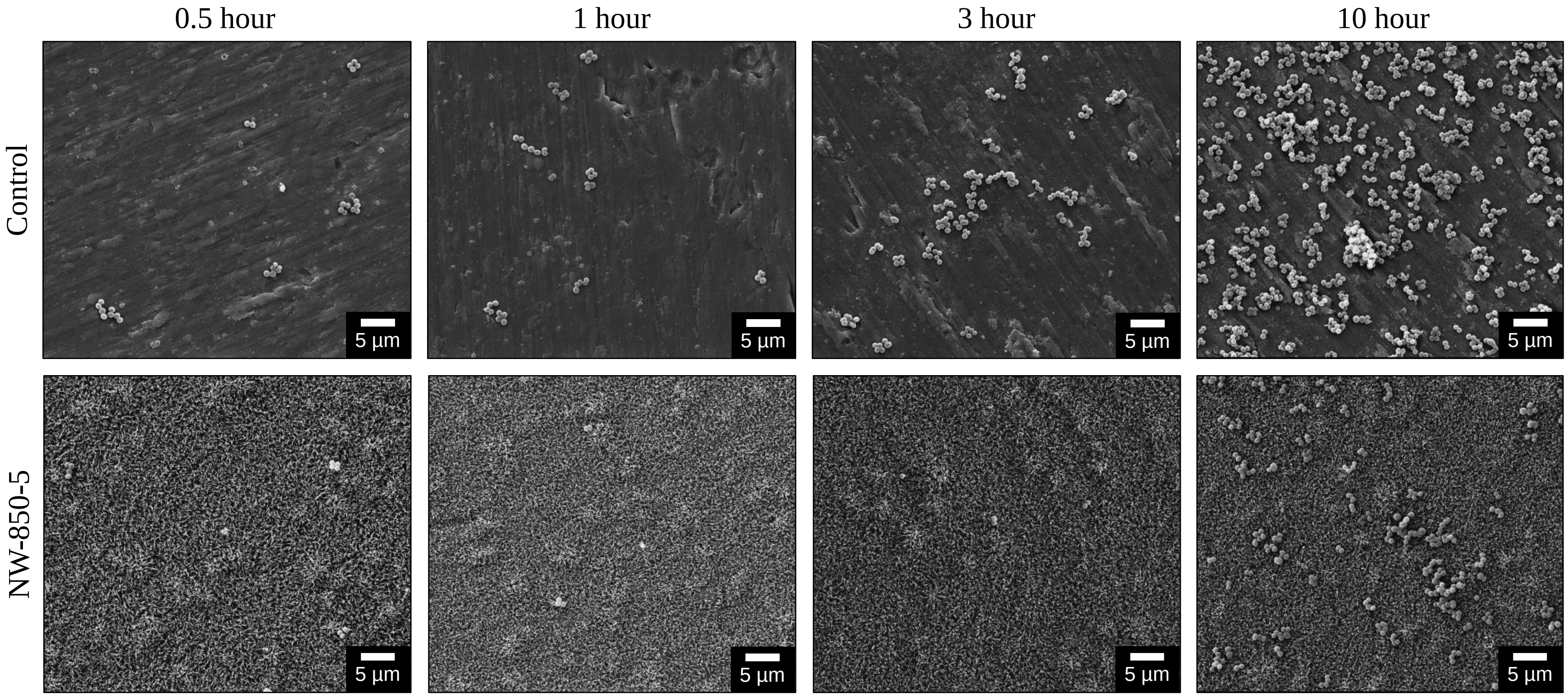

Supplementary Figure 6 │ Determination of *S. aureus* growth on TiO_2 nanopillar surface NW-850-5. Low magnification SEM micrographs were acquired of *S. aureus* on control and NW-850-5 after 0.5-, 1-, 3- and 10-hour incubation. Scale bar = 5 µm. Micrographs are representative of three independent surfaces (n=3).

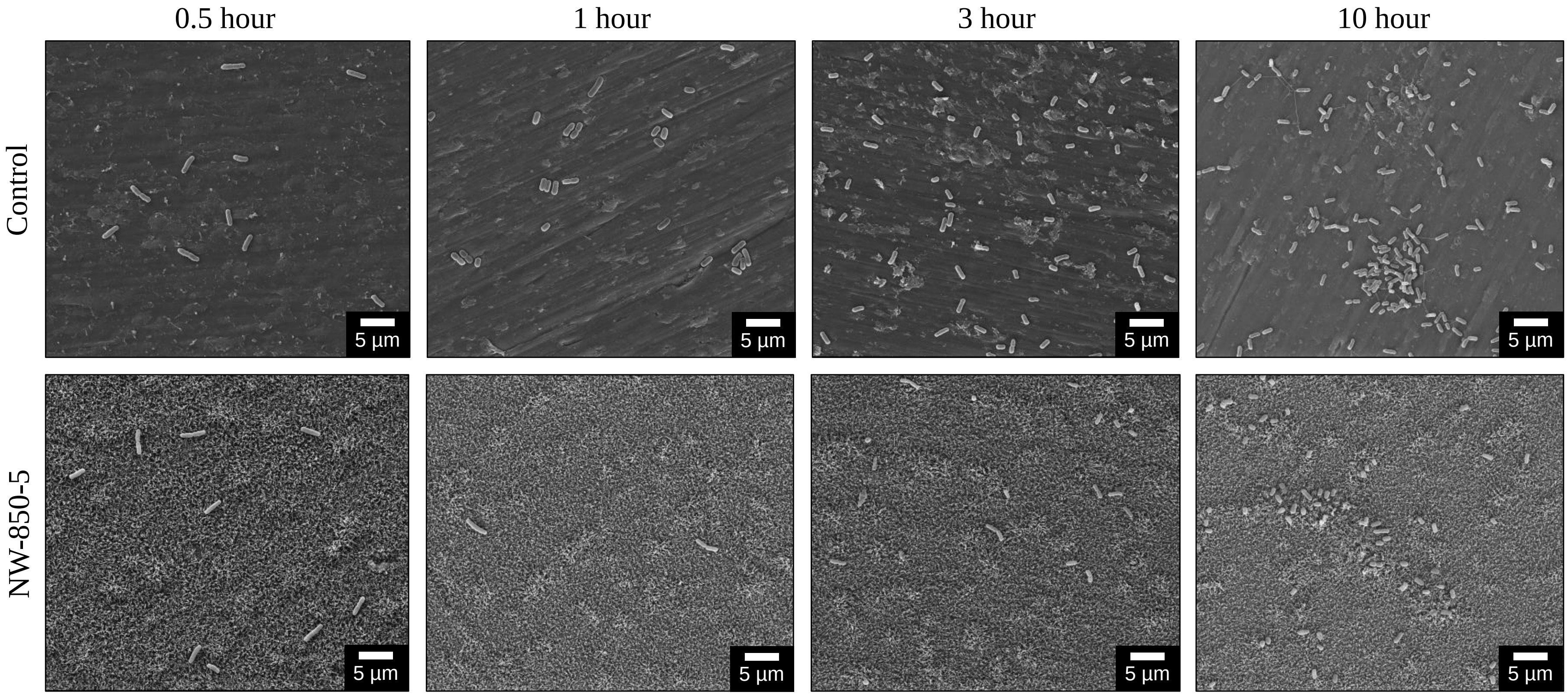

Supplementary Figure 7 | Determination of *E. coli* growth on TiO_2 nanopillar surface NW-850-5. Low magnification SEM micrographs were acquired of *E. coli* on control and NW-850-5 after 0.5-, 1-, 3- and 10-hour incubation. Scale bar = 5 µm. Micrographs are representative of three independent surfaces (n=3).

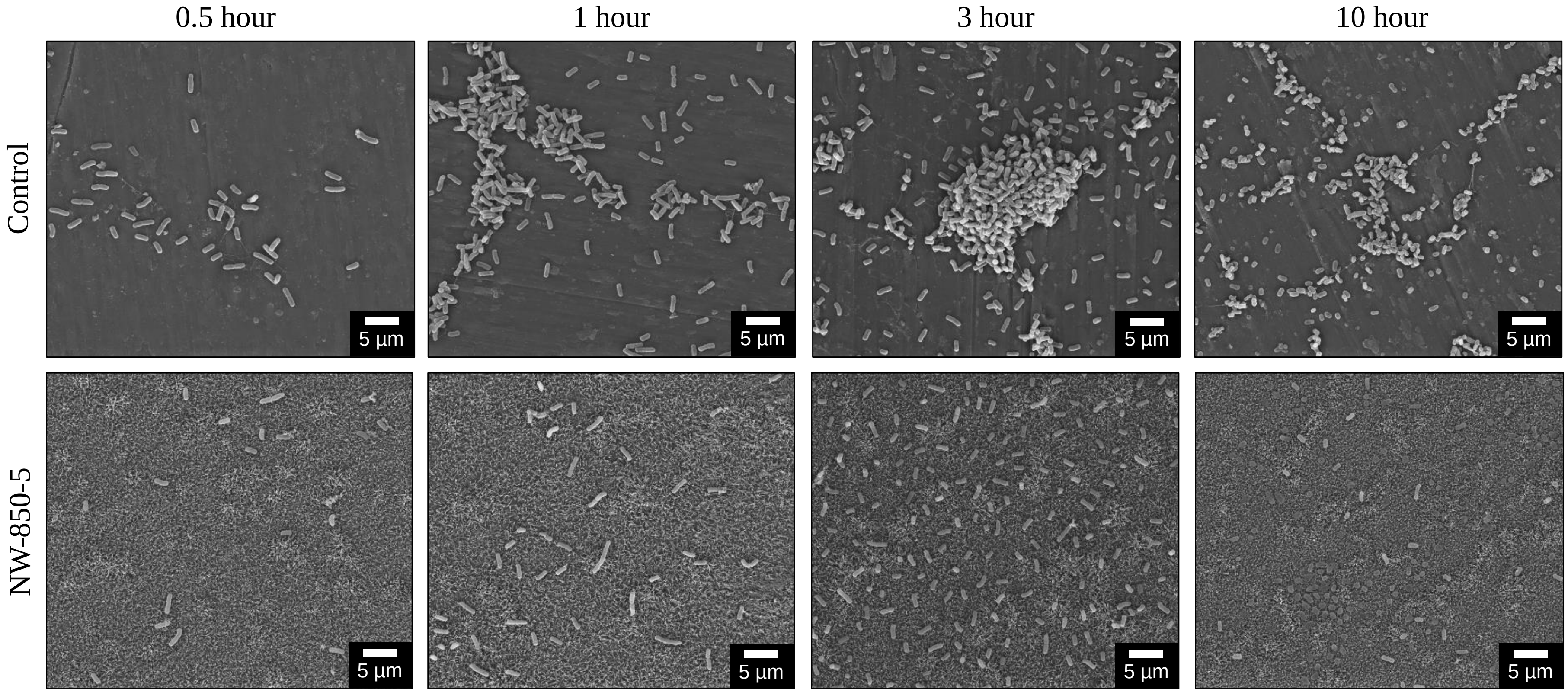

Supplementary Figure 8│Determination of *K. pneumoniae* growth on TiO_2 nanopillar surface NW-850-5. Low magnification SEM micrographs were acquired of *K. pneumoniae* on control and NW-850-5 after 0.5-, 1-, 3- and 10-hour incubation. Scale bar = 5 µm. Micrographs are representative of three independent surfaces (n=3).

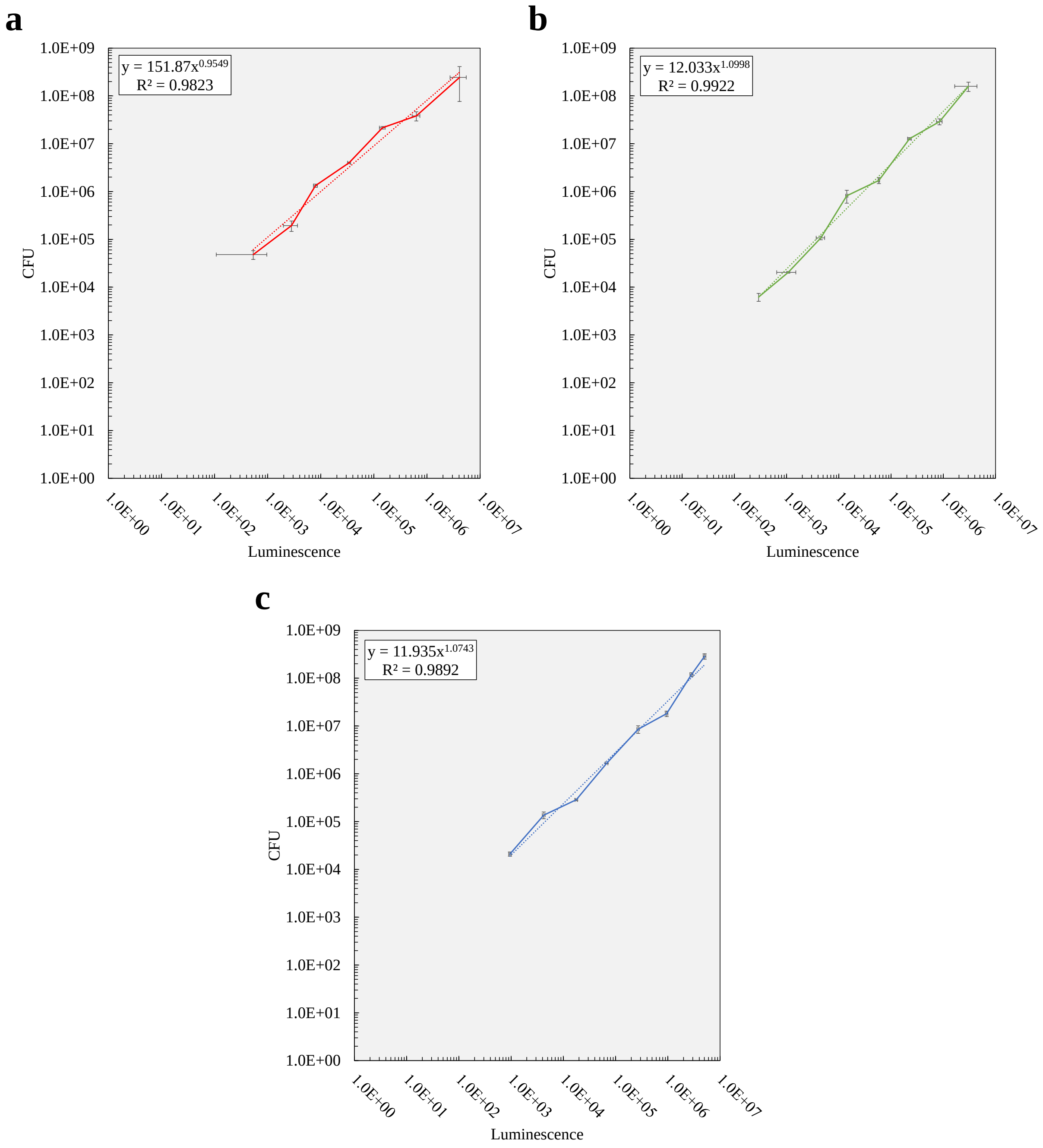

Supplementary Figure 9 | Standard curves for correlation of luminescence with CFU. Bacterial viability on TiO_2 nanopillar surfaces was semi-quantified by converting raw luminescence data (recorded from BacTiter-Glo experiments) into CFU. (a) *S. aureus*, (b) *E. coli*, (c) *K. pneumoniae*. Displayed on each graph is the corresponding equation and coefficient of determination (R^2). Values are given as mean ± standard deviation and are representative of three independent experimental replicates (n=3), performed in triplicate.

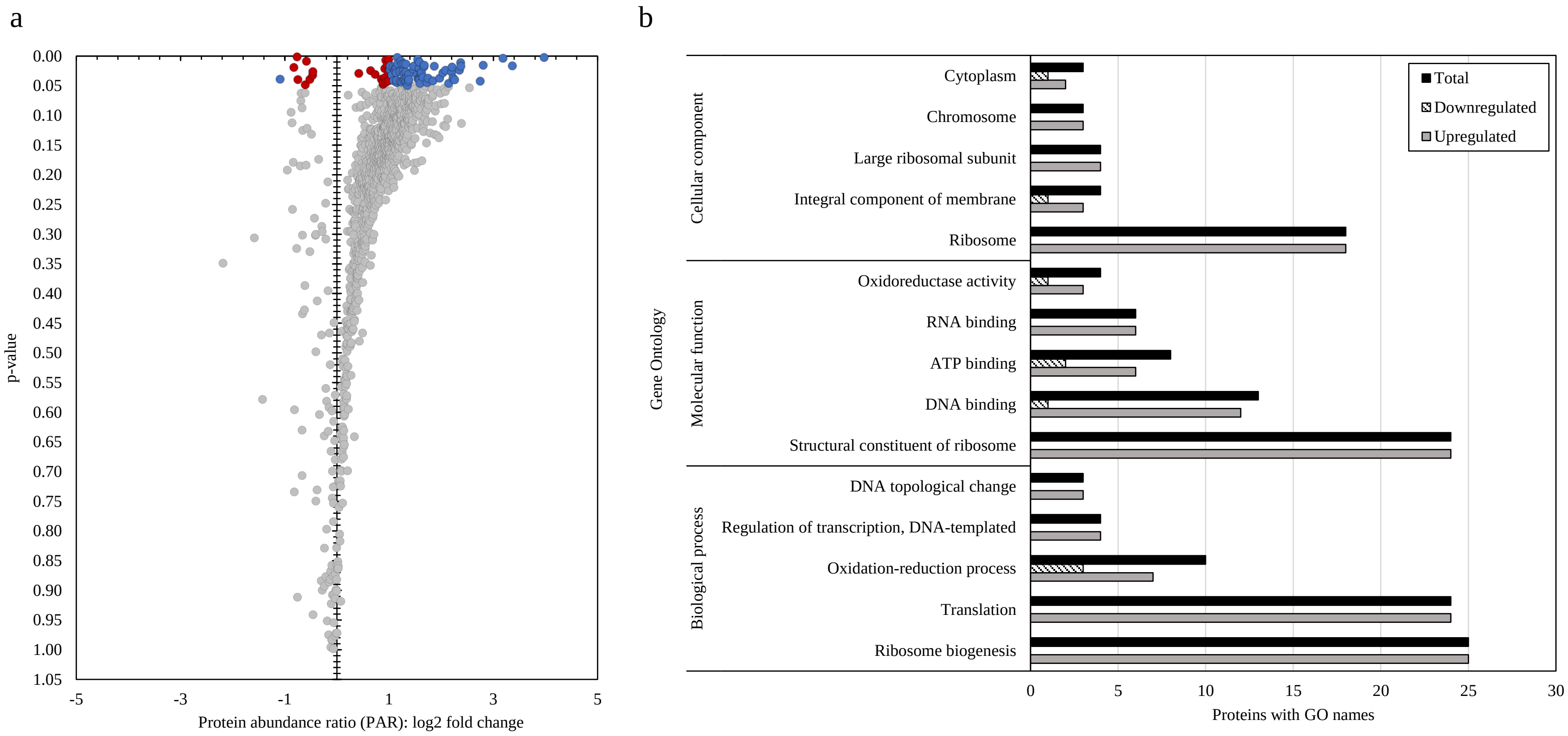

Supplementary Figure 10│Determining the *S. aureus* proteomic response to nanopillar surface NW-850-5. To investigate global physiological changes at the protein level in *S. aureus*, the abundance of cellular proteins was determined by TMT quantitative proteomic analysis, in the presence or absence of TiO_2 nanopillar surface NW-850-5. Volcano plot (a) shows *S. aureus* proteins identified from Sequest searches against the Uniprot *S. aureus* database (5% FDR); proteins that had not changed significantly between the control and nanopillar group are displayed in grey, whereas proteins showing significant abundance changes are highlighted by red (less than 2-fold changes) and blue (greater than or equal to 2-fold changes) points. *S. aureus* DEPs were then categorised using gene ontology (GO) enrichment analysis, with the five most common GO annotations for up- and down-regulated proteins according to Level 1 gene ontology (Biological Process, Cellular Component and Molecular Function) indicated (b).

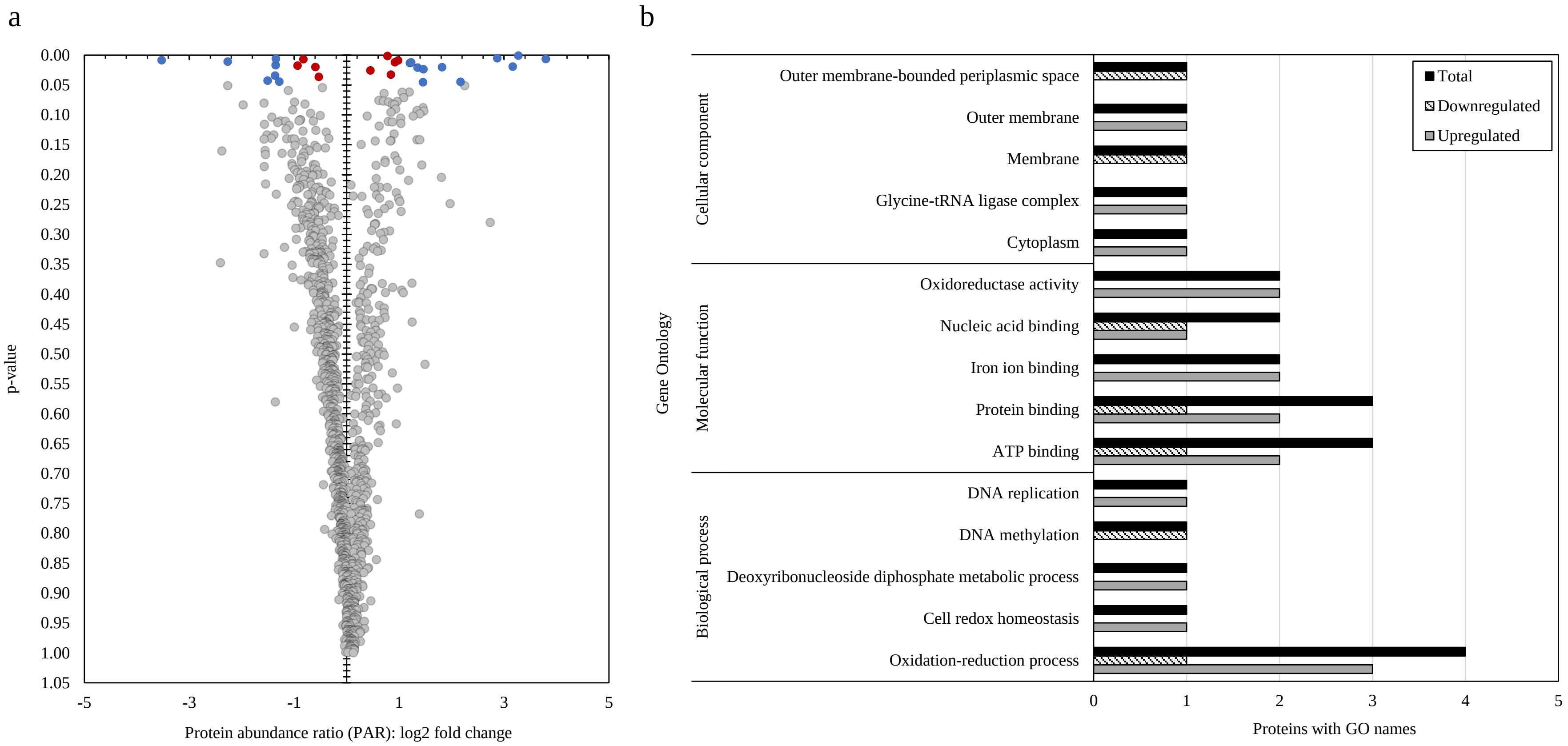

Supplementary Figure 11 | Determining the *E. coli* proteomic response to nanopillar surface NW-850-5. To investigate global physiological changes at the protein level in *E. coli*, the abundance of cellular proteins was determined by TMT quantitative proteomic analysis, in the presence or absence of TiO_2 nanopillar surface NW-850-5. Volcano plot (a) shows *E. coli* proteins identified from Sequest searches against the Uniprot *E. coli* database (5% FDR); proteins that had not changed significantly between the control and nanopillar group are displayed in grey, whereas proteins showing significant abundance changes are highlighted by red (less than 2-fold changes) and blue (greater than or equal to 2-fold changes) points. *E. coli* DEPs were then categorised using gene ontology (GO) enrichment analysis, with the five most common GO annotations for up- and down-regulated proteins according to Level 1 gene ontology (Biological Process, Cellular Component and Molecular Function) indicated (b).

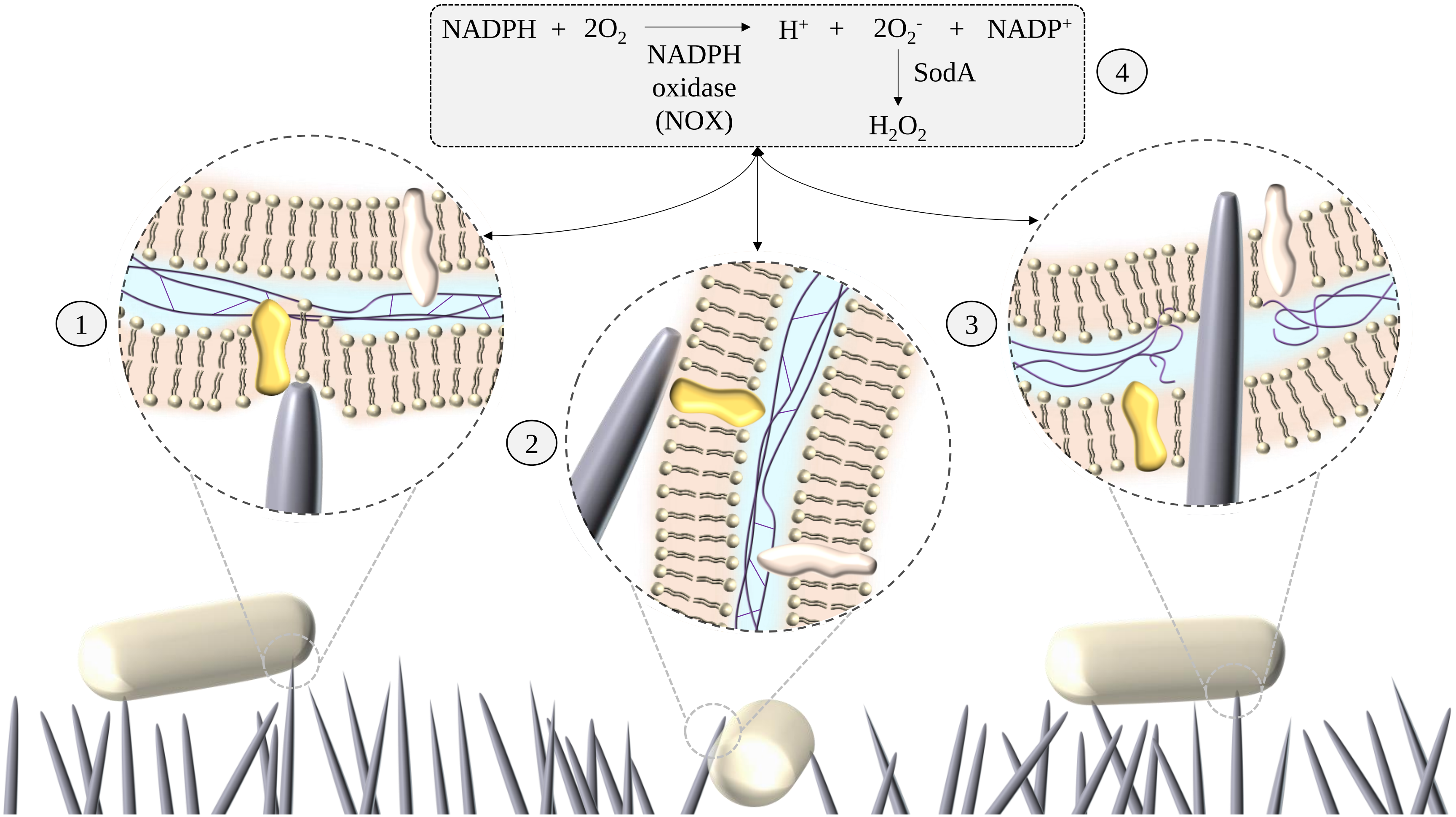

Supplementary Figure 12 | Proposed antibacterial mechanisms of TiO_2 nanopillar surface. Adhesion of bacteria to TiO_2 nanopillars can lead to envelope deformation (1). Alternatively, if the spacing of nanopillars is greater than the dimensions of the bacteria, it is possible for cells to adhere between nanopillars (2). Both outcomes can lead to cell impedance and impaired replication. When the elastic limit of the bacterial envelope is exceeded by the applied force of a nanopillar, deformation may lead to penetration of the cell envelope (3). Physical contact between bacteria and TiO_2 nanopillars can also result in an oxidative stress response induced by the production of ROS (4). The accumulation of ROS is anticipated to lead to reduced bacterial growth and increased permeability of the cell envelope.

Supplementary References

1. Spurr, R. A. & Myers, H. Quantitative Analysis of Anatase-Rutile Mixtures with an X-Ray Diffractometer. *Anal. Chem.* **29**, 760–762 (1957).
