# Supplementary material for: Antibacterial effects of nanopillar surfaces are mediated by cell impedance, penetration and induction of oxidative stress
Source: Nat Commun. 2020 Apr 2;11:1626. doi: 10.1038/s41467-020-15471-x (PMC7118135; doi:10.1038/s41467-020-15471-x)
Supplement: Supplementary file 3 — Reporting Summary [file 41467_2020_15471_MOESM3_ESM.pdf]

nature research

Corresponding author(s): Bo Su, Angela H. Nobbs

Last updated by author(s): Feb 24, 2020

Reporting Summary

Nature Research wishes to improve the reproducibility of the work that we publish. This form provides structure for consistency and transparency in reporting. For further information on Nature Research policies, see Authors & Referees and the Editorial Policy Checklist.

Statistics

For all statistical analyses, confirm that the following items are present in the figure legend, table legend, main text, or Methods section.

n/a	Confirmed	
☐	☒	The exact sample size (*n*) for each experimental group/condition, given as a discrete number and unit of measurement
☐	☒	A statement on whether measurements were taken from distinct samples or whether the same sample was measured repeatedly
☐	☒	The statistical test(s) used AND whether they are one- or two-sided<br>*Only common tests should be described solely by name; describe more complex techniques in the Methods section.*
☐	☒	A description of all covariates tested
☐	☒	A description of any assumptions or corrections, such as tests of normality and adjustment for multiple comparisons
☐	☒	A full description of the statistical parameters including central tendency (e.g. means) or other basic estimates (e.g. regression coefficient) AND variation (e.g. standard deviation) or associated estimates of uncertainty (e.g. confidence intervals)
☒	☐	For null hypothesis testing, the test statistic (e.g. *F*, *t*, *r*) with confidence intervals, effect sizes, degrees of freedom and *P* value noted<br>*Give P values as exact values whenever suitable.*
☒	☐	For Bayesian analysis, information on the choice of priors and Markov chain Monte Carlo settings
☒	☐	For hierarchical and complex designs, identification of the appropriate level for tests and full reporting of outcomes
☒	☐	Estimates of effect sizes (e.g. Cohen's *d*, Pearson's *r*), indicating how they were calculated

Our web collection on statistics for biologists contains articles on many of the points above.

Software and code

Policy information about availability of computer code

Data collection	Data collection for BacTiter-Glo, RealTime-Glo and ROS-Glo assays was performed using Magellan software v7.1 (Tecan). Acquisition of tilt series data was performed using Tomography 4 software (ThermoFisher). Tomograms were aligned and constructed using the ETomo package (IMOD v4.9). Acquisition of FIB-SEM data was performed using Auto Slice and View 4 software (ThermoFisher). For proteomic analysis, all spectra were acquired using an Orbitrap Fusion Tribrid mass spectrometer controlled by Xcalibur 2.0 software (Thermo Scientific) and operated in data-dependent acquisition mode using an SPS-MS3 workflow.
Data analysis	GIXD image data were converted into 1D profiles by azimuthal integration using a pyFAI (0.18.0a.0) package. Segmentation of tomograms and FIB-SEM data was performed using Avizo v9.7.0 (ThermoFisher). The raw proteomic data files were processed and quantified using Proteome Discoverer software v2.1 and searched against the UniProt S. aureus (strain Newman) database (downloaded October 2018; 2584 entries) or the Uniprot E. coli (strain K12) database (downloaded February 2019; 4469 entries) using the SEQUEST algorithm. Blast2Go software (version 5.2) was used to categorise S. aureus and E. coli DEPs based on GO terms; DEPs were grouped by Level 2 GO. Protein–protein interactions were investigated using the functional protein association network tool (STRING version 11) within Cytoscape (version 3.7.2). Statistical analyses were performed using IBM SPSS statistical package (version 25). Fiji (v1.52p) software was used to visualise tomograms.

For manuscripts utilizing custom algorithms or software that are central to the research but not yet described in published literature, software must be made available to editors/reviewers. We strongly encourage code deposition in a community repository (e.g. GitHub). See the Nature Research guidelines for submitting code & software for further information.

Data

Policy information about availability of data

All manuscripts must include a data availability statement. This statement should provide the following information, where applicable:

- Accession codes, unique identifiers, or web links for publicly available datasets
- A list of figures that have associated raw data
- A description of any restrictions on data availability

The data that support the findings of this study are available from the corresponding author upon reasonable request. The mass spectrometry proteomics data have been deposited to the ProteomeXchange Consortium via the PRIDE partner repository with the dataset identifier PXD017078. UniProt S. aureus (strain Newman) database (downloaded October 2018; 2584 entries, Proteome ID: UP000006386) or the Uniprot E. coli (strain K12) database (downloaded February 2019; 4469 entries, Proteome ID: UP000000625).

Field-specific reporting

Please select the one below that is the best fit for your research. If you are not sure, read the appropriate sections before making your selection.

☒ Life sciences ☐ Behavioural & social sciences ☐ Ecological, evolutionary & environmental sciences

For a reference copy of the document with all sections, see nature.com/documents/nr-reporting-summary-flat.pdf

Life sciences study design

All studies must disclose on these points even when the disclosure is negative.

Sample size	Sample size was determined based on previous experience. Experimental replicates were performed in duplicate or triplicate to identify intra-sample variation.
Data exclusions	Data relating to S. epidermidis and surface NW-715-45 were excluded from the analyses at the reviewers request due to partial imaging data.
Replication	Unless otherwise stated, at least three independent experimental replicates (n=3) were performed to identify inter-experimental variation and to ensure reproducibility.
Randomization	Two surface types (Control or NW-850-5) were tested. These were randomly allocated across experimental groups using the three bacterial species (S. aureus, E. coli or K. pneumoniae).
Blinding	Due to differences in appearance between the two surface types tested (Control or NW-850-5), blinding was not possible.

Reporting for specific materials, systems and methods

We require information from authors about some types of materials, experimental systems and methods used in many studies. Here, indicate whether each material, system or method listed is relevant to your study. If you are not sure if a list item applies to your research, read the appropriate section before selecting a response.

Materials & experimental systems

n/a	Involved in the study	
☒	☐	Antibodies
☒	☐	Eukaryotic cell lines
☒	☐	Palaeontology
☒	☐	Animals and other organisms
☒	☐	Human research participants
☒	☐	Clinical data

Methods

n/a	Involved in the study	
☒	☐	ChIP-seq
☒	☐	Flow cytometry
☒	☐	MRI-based neuroimaging
